# Probiotic: is diet part of the efficacy equation?

**DOI:** 10.1080/19490976.2023.2222438

**Published:** 2023-06-28

**Authors:** Héloïse Rytter, Emilie Combet, Benoit Chassaing

**Affiliations:** a INSERM U1016, Team “Mucosal Microbiota in Chronic Inflammatory diseases”, CNRS, UMR10 8104, Université Paris Cité, Paris, France; b Human Nutrition, School of Medicine, college of Medical, Veterinary and Life Sciences, University of Glasgow, Glasgow, UK

**Keywords:** Probiotic, diet, microbiota, metabolic disorder, personalized response

## Abstract

Discovered at the beginning of the 20^th^ century by Nobel laureate Élie Metchnikoff, probiotics have more recently emerged as a potential noninvasive therapeutic approach for the treatment of various chronic diseases. However, recent population-based clinical studies suggest that probiotics are often ineffective and may even exhibit potential deleterious effects. Hence, a deeper molecular understanding of strain-specific beneficial effects, together with the identification of endogenous/exogenous factors modulating probiotic efficacy, is needed. The lack of consistency in probiotic efficacy, together with the observation that numerous preclinical findings on probiotics are not translating once applied to humans through clinical trials, suggests a central role for environmental factors, such as dietary patterns, in probiotic efficacy. Two recent studies have been instrumental in filling this knowledge gap, defining the role played by diet in probiotic efficacy on metabolic deregulations in both mouse models and humans .

## GRAPHICAL ABSTRACT

**Figure:**
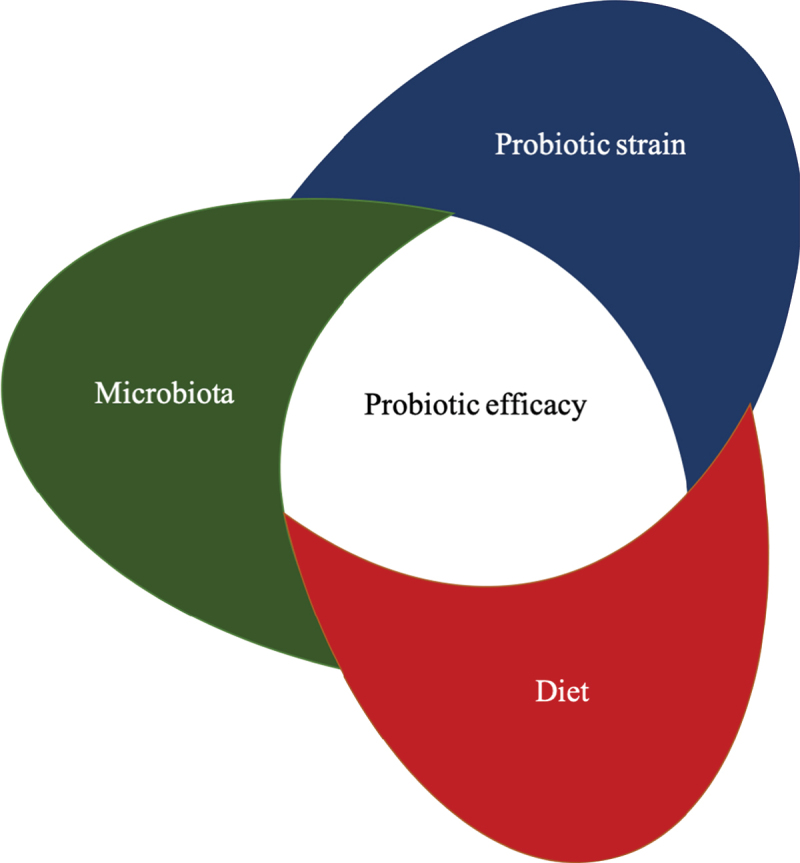

## Main

With the increased incidence of several chronic inflammatory diseases, including inflammatory bowel disease and metabolic deregulations, numerous drug-based pharmacological therapies have emerged. Moreover, while there is still no current clinical use, probiotics, described as “*live microorganisms which when administered in adequate amount confer a health benefit on the host*”,^1^ could offer innovative therapeutic venues in select disease conditions. Among them, next-generation probiotics, defined as microbiota-derived functional microbes with beneficial properties, are gaining research interest. For example, *Faecalibacterium prausnitzii* is greater in abundance in Crohn’s disease patients with a lower risk of postoperative recurrence, and its administration in animal models has been shown to dampen induced chronic intestinal inflammation.^2^ Another example further highlighting the growing interest in probiotics relates to *Akkermansia muciniphila*, with the observation that exogenous administration of this bacterium promotes intestinal and metabolic health in both animal models and in pilot human randomized controlled trial (RCT).^3,4^

Such observations concomitantly drove growing interest from industrial actors in developing new probiotics, and numerous research lines are currently investigating their specific characteristics and impacts on human health. Despite this fast-growing interest, molecular mechanisms underpinning probiotic-mediated beneficial impacts on the host’s health remain poorly understood. A recent pioneering study from Eran Elinav’s group demonstrated that human consumption of a specific commercial probiotic preparation that included 11 strains results in highly individualized colonization of the intestinal mucosa.^5^ Interestingly, basal intestinal microbiota, together with select host factors, appears to differentiate individuals as *permissive* versus *resistant* to probiotic colonization. Moreover, another study from this group suggested that probiotics regimen is not the most effective approach for microbiota reconstitution following antibiotic treatment, with the observation that probiotic consumption significantly delayed the restoration of a “normal” microbiota richness.^6^ Altogether, these data highlight the pressing need for in-depth characterization of factors impacting probiotic-mediated modulation of intestinal and metabolic health. Two recent *Gut Microbes* articles made significant progress in this era by highlighting the important role played by diet in probiotic efficacy on host metabolism, in both mice models and in a human RCT.

In an 18-week double-blinded, placebo-controlled study in adults with metabolic deregulations, Wastyk *et al*. report a high level of inter-individual variations in the beneficial effects driven by probiotic intake.^7^ Compared to the control group, only a subset of 10 out of 26 participants who received a probiotic cocktail containing three lactic acid bacteria strains (*Limosilactobacillus reuteri* NCIMB 30,242, *Lactiplantibacillus plantarum* UALp−05™, and *Bifidobacterium animalis* subsp. *lactis*B420™) exhibited decreased triglycerides concentration. Metabolic markers either did not improve in other participants or even slightly worsened, further emphasizing how essential it is to characterize such interindividual variability prior to proposing any probiotic regimen to a given patient. Moreover, authors identified, in a secondary exploratory analysis, that the difference between responder and non-responders could be driven by their habitual diet. Indeed, the authors observed a different intake of seven nutrients, including added sugar, lactose or sucrose, by probiotic-responders compared to probiotic-non-responders. Hence, this suggests that consumption of these diet-derived components could mediate sensitivity to probiotic-driven modulation of metabolic health, either directly or indirectly through modulation of the intestinal microbiota. Moreover, circulating levels of homovanillic acid (HVA) could differentiate responders and non-responders with a 71% accuracy. HVA is one of the numerous colonic metabolites produced from the fermentation of various dietary (poly)phenolics, abundant bioactives present in plant foods, with a recognized prebiotic activity. With the previous observation that diet-derived flavonols could significantly elevate HVA excretion,^8^ dietary habits between responder and non-responders could be at play in modulating such HVA serum level and probiotic efficacy. To conclude, this study identifies diet as a key modulating factor of probiotic efficacy in humans with metabolic deregulations. Of note, inherent to human-based RCT, other factors could play a role in such probiotic efficacy, such as genetic predisposition, and larger RCT appears needed to better characterize factors modulating probiotic efficacy, in humans, for future improvement of probiotic efficacy and/or personalized probiotic regimen.

Importantly, another study in the same *Gut Microbes* issue investigated, through a mice-based model, potential factors involved in probiotic efficacy, including genetical factors.^9^ In this study, Ida Larsen *et al*. evaluated the potential effect of two probiotic strains (*Limosilactobacillus reuteri* DSM 32,910 and *Lacticaseibacillus paracasei* DSM 32,851) on inbred mice fed with either a classical high-fat diet (HFD) or a customized diet mimicking fast-food regimen (FFMD) designed to exacerbate NAFLD. Although diets were energy-matched, they drove significantly different detrimental phenotypes following a 12-week exposure, as elegantly deeply characterized by the authors. Both HFD and FFMD-induced obesity and compromised glucoregulatory capacity, but FFMD led to enhanced ectopic fat distribution to the visceral fat as well as the liver. More importantly, this study reports that a given probiotic strain could impact aspects of metabolic disorders in a diet-dependent manner. Indeed, the authors observed that *L. paracasei* reduced obesity development with decreased NAFLD in HFD, but not in FFMD, fed mice. On the other hand, *L. reuteri* administration was sufficient to improve glucose homeostasis, reduce NAFLD development and increase distal gut bile acid levels associated with changes in predicted functions of the gut microbiota only in FFMD-fed mice. Hence, these data suggest that probiotic efficacy of two LAB strains is highly dependent on the experimental obesogenic diets being used to induce metabolic deregulations. Furthermore, Ida Larsen *et al*. observed an increase in *Proteobacteria* phylum abundances in FFMD-fed mice, which has been linked to epithelial damage related to anaerobiosis disruption, but the tested probiotic strains had negligible impact on microbiota composition. Hence, the exact role played by the intestinal microbiome in driving such diet-mediated probiotic efficacy remains to be characterized.

To conclude, these two reports highlight that dietary habits potentially impact probiotic efficacy in obesity-related dysmetabolism, in both mice models and humans. Hence, these observations suggest that the beneficial effects associated with probiotic intake could be mischaracterized in clinical trials lacking careful and comprehensive investigation of dietary habits. Both studies therefore constitute an important methodological framework and reference for future studies investigating probiotic efficacy in metabolic disorders, as well as in other chronic diseases with an inflammatory component. Further studies are needed to determine, for instance, whether a non-responsive state of a given probiotics is stable or could be beneficially manipulated by targeted dietary modification. It was, for example, previously observed that various carbohydrate (tested as mono- and disaccharide glucose, fructose, sucrose, lactose, galactose, and xylose as carbon sources) can enhance growth and survival of the probiotic *Lactococcus lactis*^10^ *in vitro*, further highlighting the potential of diet-probiotic interaction. Hence, in-depth mapping of dietary factors promoting the beneficial effects of any given probiotic could allow the establishment of dietary recommendations before and during probiotic regimen. The exact role played by the intestinal microbiota in mitigating probiotics effectiveness remains to be fully determined. It can be hypothesized that various dietary factors indirectly modulate probiotic efficacy through modulation of the intestinal microbiota ecosystem composition and/or function. It was, for example, previously reported that the abundance of various microbiota members can favor the beneficial action of a given dietary intervention. For example, Karine Clément’s group reported that patients with either overweight or obesity and a higher baseline *A. muciniphila* exhibited a more marked improvement in clinical parameters, including total and LDL cholesterol, after caloric restriction compared to patients with lower baseline *A. muciniphila*.^11^ Similarly, probiotics’ efficacy could depend on several microbiota actors, and the characterization of the triangular relationship between diet, the intestinal microbiota and probiotics are needed to optimize probiotic efficacy on intestinal and metabolic health. Notwithstanding the underlying mechanism(s), these two recent studies importantly suggest that habitual diets should be carefully evaluated when investigating the effects of various probiotic regimens on intestinal and metabolic health.
